# Clinical, Biochemical, and Molecular Analyses of Medium-Chain Acyl-CoA Dehydrogenase Deficiency in Chinese Patients

**DOI:** 10.3389/fgene.2021.577046

**Published:** 2021-03-23

**Authors:** Zhuwen Gong, Lili Liang, Wenjuan Qiu, Huiwen Zhang, Jun Ye, Yu Wang, Wenjun Ji, Ting Chen, Xuefan Gu, Lianshu Han

**Affiliations:** Department of Pediatric Endocrinology/Genetics, Shanghai Institute for Pediatric Research, Xinhua Hospital, School of Medicine, Shanghai Jiao Tong University, Shanghai, China

**Keywords:** *ACADM* gene, medium-chain acyl-CoA dehydrogenase deficiency, newborn screening, octanoylcarnitine, tandem mass spectrometry

## Abstract

**Objective:**

Medium-chain acyl-CoA dehydrogenase deficiency (MCADD) is a rare inherited metabolic disorder of fatty acid β-oxidation. The present study aimed to evaluate clinical and biochemical manifestations, and the mutation spectrum of this disorder in a large cohort of Chinese patients.

**Methods:**

A total of 24 patients were enrolled, and blood acylcarnitine and urinary organic acid levels were measured by tandem mass spectrometry and gas chromatography–mass spectrometry (GC–MS), respectively. Mutations in the *ACADM* gene were detected by Sanger or next-generation sequencing. Clinical progression, acylcarnitine spectra, and mutations were analyzed and described in detail.

**Results:**

Among the 24 patients, six cases were diagnosed because of disease onset with symptoms such as vomiting, diarrhea, convulsion, and hypoglycemia; 18 patients without symptoms were diagnosed by newborn screening (NBS). All patients who accepted treatment after diagnosis developed normal intelligence and physique. The concentrations of octanoylcarnitine, the octanoylcarnitine/decanoylcarnitine ratio, and the octanoylcarnitine/acetylcarnitine ratio in the blood and urinary dicarboxylic acid concentrations were consistently elevated. Blood biomarkers failed to decrease after treatment. DNA sequencing revealed seven known and 17 novel mutations in the *ACADM* gene of patients. Mutation p.T150Rfs^∗^4 was most frequent, followed by p.R31C, p.F103Y, p.I223T, p.G362E, and c.387+1delG.

**Conclusion:**

Despite biochemical abnormalities, medium-chain acyl-CoA dehydrogenase deficiency showed relatively mild clinical phenotypes with low mortality and optimistic prognoses in China. NBS is crucial for early diagnosis, treatment, and prognosis.

## Introduction

Medium-chain acyl-CoA dehydrogenase deficiency (MCADD; OMIM 607008) is a rare autosomal, recessively inherited metabolic disease of mitochondrial fatty acid β-oxidation. This disorder occurs more frequently in Caucasian populations at an estimated incidence of 1/8,000–1/20,000, with 1/4,900–1/8,500 in Germany and 1:10,000–30,000 in the United States (Grosse et al., 2006; Rhead, 2006). The incidence is relatively low among Asians, for example, at about 1:100,000 in Japan (Hara et al., 2016). In China, MCADD incidence varies significantly between regions and was reported at 1/80,332–1/282,591 based on newborn screening (NBS) data (Wang et al., 2019; Yan et al., 2019; Yang et al., 2020). From 2013 to 2018, a total of 1,104,703 newborns were screened by tandem mass spectrometry (MS/MS) technology in our NBS center (Shanghai NBS Center in Xinhua Hospital), and 11 cases of MCADD were diagnosed, indicating that the incidence of MCADD in Shanghai was 1/100,428.

Medium-chain acyl-CoA dehydrogenase deficiency may elicit complex clinical symptoms and biochemical abnormalities, such as vomiting, diarrhea, lethargy, seizures, coma, hypoglycemia, and hyperammonemia, among which hypoglycemia and seizures are the most common symptoms. The clinical diagnosis of MCADD is based on biochemical parameters including plasma acylcarnitine, blood glucose, liver functioning, creatine kinase (CK), and blood lipids. Typical MCADD acylcarnitine patterns include elevated levels of hexanoylcarnitine (C6), octanoylcarnitine (C8), and decanoylcarnitine (C10) and of the ratios C8/C2 and C8/C10, especially C8. Additionally, elevated levels of urinary dicarboxylic acids may occur (Li et al., 2019). Detection of acylcarnitine by MS/MS can help identify symptomatic and asymptomatic MCADD cases. MS/MS-based NBS helped to determine an increasing number of asymptomatic MCADD patients during the neonatal period before disease onset. Owing to NBS, MCADD has become a manageable disorder with very low mortality and normal neurodevelopmental outcomes (Dobrowolski et al., 2017). The diagnosis can be confirmed by sequencing the *ACADM* gene, whose mutation spectrum also varies greatly between ethnicities.

In the present study, we enrolled 24 Chinese MCADD patients from the Department of Pediatric Endocrinology and Genetic Metabolism, Xinhua Hospital, Shanghai Jiao Tong University School of Medicine, and NBS center from 2009 to 2019. We performed a detailed retrospective chart review of the patients’ clinical data, including clinical process, metabolites, molecular diagnosis, treatment, and outcome. After this, we compared the data with that of age- and sex-matched healthy children in order to examine the clinical, biochemical, and genotype characteristics of MCADD.

## Materials and Methods

### Patients

A total of 24 Chinese MCADD patients (14 males and 10 females) were recruited at our department clinic and NBS center from 2009 to 2019. All patients were from unrelated families, except for two siblings (patients N5 and N6). MCADD diagnosis was confirmed by molecular genetic testing in 23 patients (except for patient N6). We analyzed the clinical and biochemical phenotypes and genotypes of all patients. Written informed consent was obtained from the parents of the study participants. This study was approved by the Ethics Committee of Xinhua Hospital.

### Metabolite Detection

The patients’ blood glucose, CK, blood lipids, and liver functions were measured by conventional biochemical methods. Blood levels of free carnitine and acylcarnitines including C6, C8, and C10 were detected by MS/MS (API 4000, American Bio-Systems Inc., Roanoke, VA, United States) using blood filter paper. The ratios C8/C2 and C8/C10 were calculated (Han et al., 2015). Urinary organic acids including dicarboxylic acids such as adipic acid, pimelic acid, suberic acid, azelaic acid, and sebacic acid were measured by gas chromatography–mass spectrometry (GC–MS) (QP2010; Shimadzu Limited, Kyoto, Japan) (Kimura et al., 1999; Fu et al., 2000).

### Mutation Analysis of the *ACADM* Gene

Genomic DNA was extracted from peripheral blood samples. The *ACADM* gene was subjected to Sanger or high-throughput next-generation sequencing, and mutations were identified using a normal human *ACADM* sequence as a reference (NCBI accession number NM_000016.5). The probability of pathogenicity and the functional impact of novel variations were assessed using five online tools: the 1,000 Genomes Project database^1^, SIFT^2^, Mutation Taster^3^, Human Splicing Finder^4^ (HSF), and HOPE^5^. The potential pathogenicity of nonsense and frameshift mutations was predicted using Mutation Taster, that of splicing mutations was predicted using Mutation Taster and HSF, and that of missense mutations was predicted based on Mutation Taster, SIFT, and HOPE. We predicted the effects of the observed novel mutations on the structure and functioning of the respective protein by bioinformatics. We mimicked possible structural changes in the *ACADM* protein using a preexisting structure model for the 11 novel missense mutations. The effects of mutations on protein functions were also predicted by HOPE.

### Statistical Analyses

Data that did not significantly deviate from normal distribution were tested using unpaired two-tailed *t*-test, and non-normally distributed data were tested using Mann–Whitney *U* test. Statistical analyses were performed using GraphPad Prism 5 software (GraphPad Software Inc., San Diego, CA, United States). Differences between groups were considered statistically significant when *P*-value < 0.05.

## Results

### Clinical Characteristics

#### Clinical Characteristics at Disease Onset

Detailed patient information is summarized in Table 1. There were 14 males and 10 females in the cohort. The 24 patients were aged 2 months to 10 years, with a median age of 4.77 years by December 2019. Among them, six cases were diagnosed because of disease onset with typical symptoms (P1–P6). They did not perform MS/MS-based NBS for their birthplace has not developed the MS/MS technology when the patients were born. The other 18 cases were identified by MS/MS-based NBS (patients N1–N18), among which 11 cases were identified by our NBS center. None of the 18 patients diagnosed from NBS showed signs of disease onset. After diagnosis, all the 24 patients began their treatment at an average age of 7 months (5–36 months).

**TABLE 1:** Clinical characteristics and the mutations in the *ACADM* gene of the medium-chain acyl-CoA dehydrogenase deficiency (MCADD) patients.

Case No.Sex	NS	Age of beginning to treat	Disease onset	Symptoms	Current health condition	On presentation														
						C0	C6	C8	C10	C8/C2	C8/C10	Glu	CK	ALT	AST	Suberic acid	Azelaic acid	Sebacic acid	Adipic acid	Pimelic acid
P1M	No	27 m	Yes	Convulsion coma	Hemiplegia	/	/	0.64	0.08	/	7.67	5.01	/	199	122	7.6	13.84	2.88	1	5.68
P2F	No	16 m	Yes	Diarrhea, vomiting, convulsion	Hypoglycemia	10.14	0.08	0.56	0.05	0.06	10.59	Hypogly cemia	/	41	81	113.48	7.46	88.55	62.14	12.93
P3M	No	34 m	Yes	Lethargy, hypoglycemia	Health	34.13	0.22	0.53	0.08	0.05	6.63	Hypogly cemia	/	/	/	15.2	37.13	1.91	3.85	27.08
P4M	No	15 m	Yes	Vomiting, diarrhea, convulsion	Loss of follow-up	14.38	/	0.38	/	/	/	5	1,671	158	522	/	/	/	/	/
P5M	No	7 m	Yes	Cough, lethargy	Health	2.72	0.10	0.21	0.17	0.07	1.26	/	/	30.5	74.5	45.79	1.74	1.62	5.81	6.1
P6F	No	36 m	Yes	Fever, lethargy, hypoglycemia	Health	12.79	0.23	1.30	0.10	0.19	13.54	4.6	87	13	38	/	/	/	/	/
N1M	Yes	2.7 m	No	Asymptomatic	Loss of follow-up	54.59	0.30	0.71	0.17	0.02	4.16	4.83	351	24	34	18.85	27	1.95	7.55	15.25
N2M	Yes	0.63 m	No	Asymptomatic	Loss of follow-up	16.32	0.28	1.99	0.16	0.15	12.44	4.96	159	19	27	73.53	10.98	32.09	22.38	7.79
N3M	Yes	0.8 m	No	Asymptomatic	Health	15.48	0.52	1.78	0.14	0.25	12.71	/	/	/	/	22.99	1.88	17.99	7.06	3.01
N4F	Yes	3.3 m	No	Asymptomatic	Health	34.05	0.82	4.15	1.00	0.21	4.16	4.6	105	19	27	39.94	12.12	23.91	10.65	8.74
N5F	Yes	1 m	No	Asymptomatic	Health	22.56	0.90	7.43	0.56	0.64	13.27	/	/	/	/	/	/	/	/	/
N6M	Yes	0.87 m	No	Asymptomatic	Health	24.10	0.41	2.14	0.22	0.16	9.73	/	/	/	/	/	/	/	/	/
N7M	Yes	3.6 m	No	Asymptomatic	Health	/	0.54	2.09	0.57	/	3.64	5.1	117	55	49					
N8F	Yes	0.5 m	No	Asymptomatic	Health	18.41	0.55	6.70	0.59	0.38	11.45	/	N	4	15	/	/	/	/	/
N9F	Yes	0.5 m	No	Asymptomatic	Health	13.28	0.26	1.54	0.15	0.28	10.04	3.9	131	76	103	24.85	1.66	5.12	9.07	4.66
N10F	Yes	6.5 m	No	Asymptomatic	Loss of follow up	/	0.85	2.95	0.42	0.14	7.02	/	/	/	/	75.35	11.4	19.19	14.78	7.83
N11F	Yes	1.3 m	No	Asymptomatic	Health	27.79	0.42	1.72	0.55	0.19	3.15	5.84	112	23	34	18.03	3.85	2.72	3.87	3.63
N12M	Yes	2 m	No	Asymptomatic	Health	/	1.86	11.29	0.69	0.67	16.36	5.4	84	35	43	17.02	17.71	4.73	4.59	11.83
N13M	Yes	0.5 m	No	Asymptomatic	Health	34.67	0.48	1.64	0.16	0.13	10.33	4.31	112	20	31	29.44	0.35	4.62	13.94	6.29
N14F	Yes	2.7 m	No	Asymptomatic	Health	/	0.39	3.94	0.21	/	18.76	5	73	46	46	11.6	10.04	2.76	3.39	4.69
N15M	Yes	5 m	No	Asymptomatic	Health	36.19	0.20	0.56	0.17	0.04	3.37	5.83	584	33	55	41.12	2.33	25	9.36	3.54
N16F	Yes	1 m	No	Asymptomatic	Health	/	0.51	1.10	0.31	/	3.55	N	N	N	N	3.01	6.08	0.21	2.01	3.9
N17M	Yes	1 m	No	Asymptomatic	Health	37.50	0.57	3.51	0.37	0.37	9.50	/	/	/	/	7.19	/	0.93	3.3	0.6
N18M	Yes	1.27 m	No	Asymptomatic	Health	18.79	0.53	2.48	0.20	0.16	12.48	4.63	80	44	51	42.17	0.31	1.48	6.27	4.16

*Clinical characteristics and biochemical indexes of the MCADD patients before treatment. M, male; F, female; NS, newborn screening; y, year; m, month; d, day. Reference range: Glu: 3.9–6.1 mmol/L; CK: 26–192 IU/L; ALT: 0–75 U/L; AST: 8–38 U/L; C0: 10–60 μmol/L; C6: 0.01–0.15 μmol/L; C8: 0.01–0.3 μmol/L; C10: 0.02–0.5 μmol/L; C8/C2: 0.0–0.1; C8/C10: 0.5–2.0; suberic acid: 0–10 mmol/mol creatinine; azelaic acid: 0–18 mmol/mol creatinine; sebacic acid: 0–17 mmol/mol creatinine; adipic acid: 0–15 mmol/mol creatinine; and pimelic acid: 0–15 mmol/mol creatinine.*

The six cases with acute signs of disease showed specific symptoms, including vomiting, diarrhea, lethargy, convulsion, and coma among others. The median age of disease onset was 16 months, ranging from 7 to 34 months. Hypoglycemia was confirmed at the onset of convulsions in patients P2, P3, and P6. Patients P1 and P4 showed normal blood glucose after convulsions; however, they exhibited reduced liver functions as was evident from elevated ALT and AST values. In patient P4, CK concentrations were markedly elevated at disease onset. We measured the concentrations of blood lipids including triglyceride, cholesterol, high-density lipoprotein, and low-density lipoprotein in patients N4, N7, N8, N11, N13, and N15, all of which were within the respective normal range.

Patient P5 experienced respiratory tract infection with cough and lethargy at the age of 7 months. The acylcarnitine profile was measured using MS/MS, and C0 was exposed extremely (2.72 μmol/L; normal range: 10–60 μmol/L), whereas C6, C8, and C10 were within the normal range as shown in Table 1. Therefore, the patient was clinically diagnosed with primary carnitine deficiency and was treated with 200 mg/kg/day L-carnitine. High-throughput next-generation sequencing showed two mutations in the *ADADM* gene. After 3 months of treatment, the C0 values were within the normal range (59 μmol/L), and obviously C6, C8, and the ratios C8/C2 and C8/C10 were substantially elevated (Table 2). This confirmed the MCADD diagnosis. The L-carnitine treatment was continued at a dosage of 80 mg/kg/day. Similarly, patient P2 suffered from vomiting, diarrhea, and convulsion at the age of 16 months. The carnitine spectrum, as determined by MS/MS, showed that C8 and the ratio C8/C2 were slightly elevated, whereas C6, C10, and the C8/C10 ratio were within the normal range. *ADADM* sequencing confirmed the diagnosis.

**TABLE 2:** Biochemical index after treatment and the mutations in the *ACADM* gene of the medium-chain acyl-CoA dehydrogenase deficiency patients.

Case no.	After treatment										Mutation 1	Mutation 2
	C0	C6	C8	C10	C8/C2	C8/C10	Glu	CK	ALT	AST		
P1	20.87	0.26	1.02	0.11	0.14	9.27	5.01	N	24	25	c.461T > G, p.L154W	c.668T > C, p.I223T
P2	7.74	0.17	2.88	0.27	0.54	10.67	5.23	121	11	29	c.449_452delCTGA, p.T150Rfs*4	c.308T > A, p.F103Y
P3	10.38	0.25	1.62	0.17	0.29	9.45	6	/	28	74	c.1040G > T, p.G347V	c.1085G > A, p.G362E
P4	9.88	1.75	13.38	1.00	/	13.38	/	/	/	/	c.338_341delCTTA, p.A113Vfs*36	c.554_586del, p.I185_G196delinsR
P5	59.00	1.03	4.80	0.06	0.18	78.69	5.1	/	10	32	c.449_452delCTGA, p.T150Rfs*4	c.308T > A, p.F103Y
P6	/	/	/	/	/	/	/	/	/	/	c.449_452delCTGA, p.T150Rfs*4	c.449_452delCTGA, p.T150Rfs*4
N1	75.17	0.36	0.64	0.17	0.02	3.68	/	/	/	/	c.449_452delCTGA, p.T150Rfs*4	c.709G > C, p.E237Q
N2	/	/	/	/	/	/	/	/	/	/	c.449_452delCTGA, p.T150Rfs*4	c.945+2T > G
N3	12.18	0.72	4.70	0.38	0.56	12.37	/	/	/	/	c.449_452delCTGA, p.T150Rfs*4	/
N4	10.16	0.16	0.67	0.18	0.18	3.80	4.81	166	30	30	c.91C > T, p.R31C	c.1045C > T, p.R349X
N5	25.60	0.36	1.52	0.15	0.22	10.32	/	/	/	/	c.449_452delCTGA, p.T150Rfs*4	c.970_971delinsAT, p.A324I
N6	19.49	0.29	1.11	0.14	0.13	8.08	/	/	/	/	/	/
N7	32.91	0.43	1.08	0.34	0.07	3.18	5.14	166	36	47	c.91C > T, p.R31C	c.157C > T, p.R53C
N8	15.31	0.48	2.12	0.16	0.34	13.26	/	/	/	/	c.709-1G > A	c.727C > T, p.R243X
N9	7.91	0.15	0.83	0.08	0.16	10.31	5.2	161	102	97	c.385G > A, p.G129R	c.668T > C, p.I223T
N10	17.65	0.46	1.30	0.18	0.14	7.45	5.2	225	26	50	c.387+1delG	c.587G > A, p.G196E
N11	9.02	0.13	0.66	0.06	0.11	10.89	5.73	157	19	44	c.449_452delCTGA, p.T150Rfs*4	c.449_452delCTGA, p.T150Rfs*4
N12	28.46	0.42	1.73	0.13	0.15	13.09	4.9	111	15	31	c.449_452delCTGA, p.T150Rfs*4	c.449_452delCTGA, p.T150Rfs*4
N13	20.53	0.47	2.14	0.22	0.25	9.73	4.66	/	21	35	c.905A > G, P. Y302C	c.1088A > G, p.D363G
N14	15.62	0.58	3.21	0.26	0.33	12.53	/	/	/	/	c.449_452delCTGA, p.T150Rfs*4	c.449_452delCTGA, p.T150Rfs*4
N15	14.16	0.11	0.37	0.09	0.05	4.18	4.4	139	25	49	/	c.387+1delG
N16	18.15	0.25	0.64	0.33	0.04	1.94	/	/	N	N	c.769G > A, p.E257K	c.1229T > C, p.I410T
N17	/	/	/	/	/	/	/	/	/	/	c.449_452delCTGA, p.T150Rfs*4	c.449_452delCTGA, p.T150Rfs*4
N18	/	/	/	/	/	/	5.16	/	/	/	c.624del, p.P209Qfs*30	c.1085G > A, p.G362E

*M, male; F, female; NS, newborn screening; y, year; m, month; d, day. Reference range: Glu: 3.9–6.1 mmol/L; CK: 26–192 IU/L; ALT: 0–75 U/L; AST: 8–38 U/L; C0: 10–60 μmol/L; C6: 0.01–0.15 μmol/L; C8: 0.01–0.3 μmol/L; C10: 0.02–0.5 μmol/L; C8/C2: 0.0–0.1; C8/C10: 0.5–2.0.*

In patient P6, disease onset was induced by an upper respiratory tract infection at the age of 16 months, which led to fever, convulsion, and hypoglycemia; however, the patient recovered within 1 week of glucose supplementation and anti-infective treatment. The second episode occurred with the same symptoms (fever, convulsion, and hypoglycemia) at the age of 35 months. Blood MS/MS examination at this point showed high C6, C8, the ratio C8/C2, and the ratio C8/C10. The MCADD diagnosis was confirmed, and regular treatment with L-carnitine began when the patient was 36 months old. The patient recovered and showed no more symptoms after the treatment.

#### Treatment

Treatments including oral L-carnitine and diet adjustment commenced immediately after confirming the diagnosis. The administered L-carnitine dosage was 50–200 mg/kg/day. The diet adjustment included a low-lipid diet and avoidance of fasting. During the follow-up, nine out of 24 patients (P3, N2, N3, N4, N7, N8, N9, N13, and N15) discontinued oral carnitine administration either voluntarily or because of side effects such as diarrhea, which occurred mostly within 3 months of treatment. More than half of the patients (16/24) maintained a normal diet, and only eight patients (P6, N3, N11, N12, N13, N16, N17, and N18) insisted on a dietary treatment.

#### Prognosis

The overall prognosis was optimistic. As for the current health condition by December 2019, four patients were lost from follow-up (P4, N1, N2, and N10), and 18 patients (18/24; 75%) were healthy and lived normal life without any symptoms. Only patient P2 showed intermittent fasting hypoglycemia at a frequency of two instances over the previous year, with normal intelligence. The patient regularly took carnitine and did not follow a controlled diet, which may be a cause of the instances of hypoglycemia. One patient (P1) was left with the sequela of hemiplegia due to a disease episode at the age of 27 months. The patient could not raise his left arm or leg but developed normal intelligence, normal physique, and clear speech.

### Biochemical Characteristics

As biochemical makers, blood values C6, C8, C10, the ratios C8/C2 and C8/C10, and urinary adipic acid, pimelic acid, suberic acid, azelaic acid, and sebacic acid were measured before treatment administration (Table 1). The blood values of C6, C8, and C10 and the ratios C8/C2 and C8/C10 after treatment are shown in Table 2. Most patients showed elevated C6 and C8 levels before treatment, especially C8. In contrast, C10 seemed less specific to MCADD, as these values were within the normal range in about half of the patients before treatment. Therefore, C8/C10 and C8/C2 turned out to be important ratios to identify MCADD. Similarly, urinary suberic acid concentrations were elevated in most patients before treatment, whereas adipic acid, pimelic acid, azelaic acid, and sebacic acid were elevated in only a few patients. This suggests that suberic acid is the most specific carboxylic acid marker in the urine of MCADD.

Both the statistic values of C8 (1.75, 0.21–11.29), ratio C8/C2 (0.16, 0.02–0.67), ratio C8/C10 (8.94 ± 0.98), and suberic acid (22.99, 3.01–13.50) before treatment and the statistic values of C8 (1.41, 0.37–13.38), ratio C8/C2 (0.16, 0.02–0.56), and ratio C8/C10 (10.02, 1.94–78.69) after treatment were elevated obviously than the normal range. Comparing the statistic values of C8, ratio C8/C2, and ratio C8/C10 after treatment with those before treatment, respectively, there was no statistic difference shown. It indicated that the treatment had little effect on these biochemical indexes of the disease (*P* > 0.01 each).

Comparing the biochemical markers between the 18 asymptomatic patients (N1–N18) and the six symptomatic patients (P1–P6), we found that the 18 cases identified by NBS showed higher C6 (0.52, 0.20–1.86 vs. 0.16 ± 0.04, *P* = 0.0044), C8 (2.09, 0.56–11.29 vs. 0.60 ± 0.15; *P* = 0.0013), and C10 (0.27, 0.14–1.00 vs. 0.10 ± 0.02, *P* = 0.0032) than the six clinical cases with typical symptoms; however, the ratios C8/C2 (0.25 ± 0.05 vs. 0.07, 0.05–0.19; *P* = 0.1094) and C8/C10 (9.23 ± 1.13 vs. 7.94 ± 2.06; *P* = 0.5966) did not differ significantly.

### *ACADM* Mutation Spectrum

Patients N3 and N15 showed only one mutation in the *ACADM* gene. Patient N6 was not sequenced; however, the sibling (N5) showed two mutations. All the other patients exhibited two mutations in the *ACADM* gene, which derived from both parents.

The mutation spectrum of the *ACADM* gene observed in this study is shown in Table 2; the cohort produced seven known mutations and 17 mutations which had not been previously reported in the Human Gene Mutation database. The known mutations included p.T150Rfs^∗^4 (34.8%), p.R53C (2.2%), c387+1delG (4.3%), p.L154W (2.2%), p.E253K (2.2%), p.R349^∗^ (2.2%), and p.G362E (4.3%). The 17 novel mutations included p.R31C (4.3%), p.F103Y (4.3%), p.G129R (2.2%), p.G196Q (2.2%), p.I223T (4.3%), p.E237Q (2.2%), p.Y302C (2.2%), p.A324I (2.2%), p.G347V (2.2%), p.D363G (2.2%), p.I410T (2.2%), p.R247^∗^ (2.2%), c.709-1G > A (2.2%), c.945+2T > G (2.2%), p.A113Vfs^∗^36 (2.2%), p.P209Qfs^∗^30 (2.2%), and p.I185_G196delinsR. Compound heterozygous mutations p.I223T/p.L154W (P1), p.T150Rfs^∗^4/p.F103Y (P2 and P5), p.G347V/p.G362E (P3), p.A113Vfs^∗^36/p.I185_G196delinsR (P4), and p.T150Rfs^∗^4/p.T150Rfs^∗^4 (P6) were identified in the six symptomatic cases (P1–P6). The mutations p.T150Rfs^∗^4, p.I223T, and p.G362E were detected in symptomatic patients and in asymptomatic neonates. Mutations p.L154W, p.G347V, p.F103Y, p.A113Vfs^∗^36, and p.I185_G196delinsR were detected only in symptomatic patients. In comparison, mutations p.R31C, p.R53C, c.387+1delG, p.R349^∗^, p.G129R, p.G196Q, p.E237Q, p.E253K, p.Y302C, p.A324I, p.D363G, p.I410T, p.R247^∗^, p.P209Qfs^∗^30, c.709-1G > A, and c.945+2T > G were observed only in asymptomatic neonates. Six variants occurred relatively frequently: p.T150Rfs^∗^4 (34.8%), p.R31C (4.3%), p.F103Y (4.3%), p.I223T (4.3%), p.G362E (4.3%), and c.387+1delG (4.3%). The mutation p.T150Rfs^∗^4 occurred with the highest frequency, accounting for 34.8% of all mutations.

**FIGURE 1:**
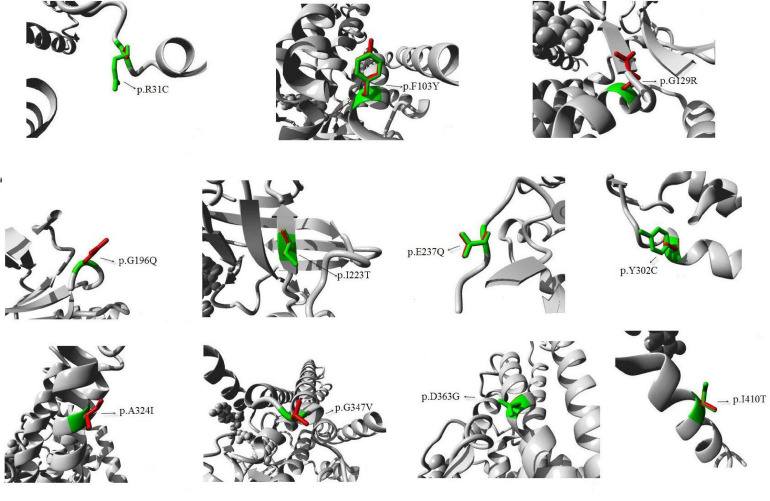
Diagram of protein structure of the novel mutations. Predicted 3D structure of human medium-chain acyl-CoA dehydrogenase. The 11 new mutations that we reported are shown. The protein is colored gray; the side chains of both the wild type and the mutant residue are shown and colored green and red, respectively.

All amino acid residues which were affected by the missense mutations were highly conserved. The effects of the 11 novel missense mutations on protein structure and functions were predicted by HOPE, as shown in Figure 1: (1) p.R31C: the wild-type Arg 31 residue and the mutant amino acids differed in size. The mutant residue (R31C) was smaller, which may lead to loss of interactions. Wild-type and mutant residues also differed regarding hydrophobicity, as the mutation introduced a more hydrophobic residue at this position. This may result in loss of hydrogen bonds and/or disturb correct folding; (2) p.F103Y: the wild type F103 residue is located in a domain that is important for binding other molecules, and it is in contact with residues in a domain that is important for protein activity. The mutation may affect this interaction and thereby disturb signal transfer from the binding domain to the activity domain; (3) p.G129R, p.G196Q, and p.G347V: the wild type of G129, G196, and G347 residues is glycine, which is the most flexible of all residues. This flexibility may be required for protein functioning, and a mutation of this residue may impede this function; (4) p.I223T: the wild-type I223 residue and mutant residue differed regarding hydrophobicity. The mutation may cause loss of hydrophobic interactions in the core of the protein; (5) p.E237Q: the residue’s charge differed between wild-type E237 and the mutant amino acid. This may disturb ionic interactions. The charge of the wild-type residue was lost due to this mutation; (6) p.Y302C: the wild-type Y302 residue was larger than the wild-type residue. The mutation will produce an empty space in the core of the protein. Wild-type and mutant residues differed regarding hydrophobicity. The mutation will cause a loss of hydrogen bonds in the core of the protein and, as a result, may disturb correct folding; (7) p.A324I: the A324 residue is located in an α-helix, and the mutation converted the wild-type residue to a residue that does not preferably occur in secondary structures of α-helices; (8) p.D363G: wild-type D363 is glycine, which is very flexible and can disturb the required rigidity of the protein at this position; and (9) p.I410T: the I410 residue is threonine, and the mutated residue is not in direct contact with a ligand; however, the mutation could affect local stability which, in turn, may interfere with the ligand contacts of one of the neighboring residues (Venselaar et al., 2010).

## Discussion

Medium-chain acyl-CoA dehydrogenase deficiency is a mitochondrial β-oxidation defect, which is potentially fatal. To identify patients with MCADD, detection of acylcarnitine by MS/MS and confirmatory mutation analysis are essential. Few Chinese cases of MCADD have been confirmed by genetic diagnosis. In the present study, we found that MCADD produced relatively mild clinical phenotypes, several biochemical abnormalities, low morbidity, and optimistic prognosis. Moreover, 17 novel mutations were identified. This study expanded the *ACADM* mutation spectrum in Chinese patients, and our results may be of importance regarding prenatal diagnosis and genetic counseling for parents of potential MCADD patients.

Medium-chain acyl-CoA dehydrogenase deficiency-positive neonates showed higher C6, C8, and C10 levels than the six clinical cases with typical symptoms, whereas the ratios C8/C2 and C8/C10 did not differ. The metabolism-related consequences of this phenomenon remain to be elucidated; however, the results indicated that C8, the ratio C8/C2, and the ratio C8/C10 are relatively disease-specific markers of MCADD. The levels of C6, C8, and C10 cannot reliably predict disease onset, whereas the ratios C8/C2 and C8/C10 appear to be more meaningful predictors. This is in line with the conclusion of Arnold who suggested that the levels of C6, C8, and C10 have no predictive value for MCADD in neonates or for the severity of later symptoms (Arnold et al., 2010). Susceptibility to certain disease phenotypes may differ between mutations; however, other genetic and environmental factors may also play an equally important role when patients are subjected to metabolic stress.

GC–MS is useful for diagnosing MCADD during acute attacks, but it is not suitable for neonatal screening (Tong et al., 2019). Regarding the organic acid spectrum as determined by GC–MS, urinary adipic acid, pimelic acid, suberic acid, azelaic acid, and sebacic acid before treatment were higher than in controls. However, urinary suberic acid concentrations were increased in most patients, while the levels of the other dicarboxylic acids were normal in most patients. This suggests that suberic acid is relatively specific to MCADD compared to the other dicarboxylic acids. This may be due to the fact that suberic acid is a metabolite of C8 (Divry et al., 1984), which is the biomarker of MCADD with the highest concentration.

The mutation spectrum of *ACADM* gene varies between ethnicities and particularly between geographically distant ethnicities. The c.985A > G mutation occurs frequently, and the c.199T > C allele is the second most frequent mutation in Caucasians (Smith et al., 2010). The mutation p.T150Rfs^∗^4 was first detected in a Korean patient (Ensenauer et al., 2005). Mutations p.T150Rfs^∗^4, p.R17H, p.G362E, p.R53C, and p.R281S accounted for approximately 60% of all alleles in a large cohort of Japanese MCADD patients, and mutation p.T150Rfs^∗^4 was the most prevalent (Tajima et al., 2016). In Chinese patients, seven mutations have been previously reported, including p.T150Rfs^∗^4, p.T121I, p.R243X, p.R53H, p.R349X, p.G362E, and c.387+1delG, with p.T150Rfs^∗^4 being the most common (Liang et al., 2015; Li et al., 2019). We observed 24 *ACADM* gene mutations, including 17 novel variants. The six most frequent variants were p.T150Rfs^∗^4, p.R31C, p.F103Y, p.I223T, p.G362E, and c.387+1delG, of which c.387+1delG was previously found in Caucasian patients, whereas the other mutations seem to be specific to East Asian patients. Mutation p.T150Rfs^∗^4, which was identified in 11 of 24 patients in the current study, was previously detected in three Korean (Woo et al., 2011) and eight Japanese patients (Purevsuren et al., 2009), but not in Caucasian patients (Sturm et al., 2012). Thus, our results confirmed that p.T150Rfs^∗^4 is the most common mutation associated with MCADD in East Asian patients. The effects of genotypes on phenotypes require further investigation, and further functional studies should be conducted to assess the effects of these novel mutations.

## Conclusion

Medium-chain acyl-CoA dehydrogenase deficiency showed relatively mild clinical phenotypes, low morbidity, and optimistic prognosis in Chinese patients. The NBS project is important for early diagnosis, treatment, and prognosis. These results will help improve our understanding of the genetic background and clinical manifestation of MCADD in Chinese patients.

## Data Availability Statement

The datasets presented in this study can be found in online repositories “Leiden Open Variation Database, LOVD,” (https://databases.lovd.nl) with the accession number 00335784–00335807.

## Ethics Statement

The studies involving human participants were reviewed and approved by the Ethics Committee of Xinhua Hospital Affiliated to Shanghai Jiao Tong University School of Medicine (Approval number: XHEC-D-2020-098). Written informed consent to participate in this study was provided by the participants’ legal guardian/next of kin.

## Author Contributions

ZG contributed to reorganizing and analyzing the clinical data of the patients and drafting of the manuscript. LL as the doctor of many of these patients, contributed to reorganizing and analyzing the clinical data of the patients and revised the manuscript. WQ, HZ, JY, and XG contributed to collecting and treating the patients and providing the clinical data. YW contributed to the gene variation analysis. WJ contributed to the detection of blood acylcarnitines of the patients by tandem mass spectrometry. TC contributed to the detection of urinary organic acids of the patients by gas chromatography–mass spectrometry. LH as the doctor of most of the patients, contributed to designing the research, treating the patients, providing the clinical data, and revising the manuscript. All authors contributed to the article and approved the submitted version.

## Conflict of Interest

The authors declare that the research was conducted in the absence of any commercial or financial relationships that could be construed as a potential conflict of interest.

## Footnotes

1 http://browser.1000genomes.org/index.html

2 http://sift.jcvi.org

3 http://www.mutationtaster.org/

4 http://www.umd.be/HSF/

5 https://www3.cmbi.umcn.nl/hope
